# An Effective DNA Methylation Biomarker Screening Mechanism for Amyotrophic Lateral Sclerosis (ALS) Based on Comorbidities and Gene Function Analysis

**DOI:** 10.3390/bioengineering11101020

**Published:** 2024-10-12

**Authors:** Cing-Han Yang, Jhen-Li Huang, Li-Kai Tsai, David Taniar, Tun-Wen Pai

**Affiliations:** 1 Department of Computer Science and Engineering, National Taiwan Ocean University, Keelung City 202301, Taiwan; icjh3105@yahoo.com.tw (C.-H.Y.); jlh110147@gmail.com (J.-L.H.); 2 Department of Neurology, National Taiwan University Hospital and National Taiwan University College of Medicine, Taipei City 100229, Taiwan; milikai@ntuh.gov.tw; 3 Faculty of Information Technology, Monash University, Clayton, VIC 3800, Australia; david.taniar@monash.edu; 4 Department of Computer Science and Information Engineering, National Taipei University of Technology, Taipei 106344, Taiwan

**Keywords:** bioinformatics, biomarker screening platform, DNA methylation, comorbidity, amyotrophic lateral sclerosis (ALS)

## Abstract

This study used epigenomic methylation differential expression analysis to identify primary biomarkers in patients with amyotrophic lateral sclerosis (ALS). We combined electronic medical record datasets from MIMIC-IV (United States) and NHIRD (Taiwan) to explore ALS comorbidities in depth and discover any comorbidity-related biomarkers. We also applied word2vec to these two clinical diagnostic medical databases to measure similarities between ALS and other similar diseases and evaluated the statistical assessment of the odds ratio to discover significant comorbidities for ALS subjects. Important and representative DNA methylation biomarker candidates could be effectively selected by cross-comparing similar diseases to ALS, comorbidity-related genes, and differentially expressed methylation loci for ALS subjects. The screened epigenomic and comorbidity-related biomarkers were clustered based on their genetic functions. The candidate DNA methylation biomarkers associated with ALS were comprehensively discovered. Gene ontology annotations were then applied to analyze and cluster the candidate biomarkers into three different groups based on gene function annotations. The results showed that a potential testing kit for ALS detection can be composed of *SOD3*, *CACNA1H*, and *ERBB4* for effective early screening of ALS using blood samples. By developing an effective DNA methylation biomarker screening mechanism, early detection and prophylactic treatment of high-risk ALS patients can be achieved.

## 1. Introduction

Neurodegenerative diseases refer to hereditary or sporadic conditions characterized by the gradual and progressive loss of neuronal structure or function, including neuronal death. Neurodegenerative disorders, such as Alzheimer’s disease (AD), Parkinson’s disease (PD), and amyotrophic lateral sclerosis (ALS), also known as Lou Gehrig’s disease, affect millions of people around the world [1]. Unfortunately, the pathogenesis of these chronic neurodegenerative diseases is not fully understood, and current treatments do not stop or slow down the progression of these pathological conditions. ALS is an adult-onset neurological disorder. Patients experience progressive degeneration of motor neurons in the spinal cord, brainstem, and cortex, leading to general paralysis, respiratory failure, and death. Clinically, patients can present with bulbar onset (25%), limb onset (70%), or trunk/respiratory onset (5%), followed by subsequent spread to adjacent regions. Based on where the onset is characterized (for example, spinal or bulbar), patients may survive for 2–10 years [2,3,4].

Early detection of the onset of neurodegeneration is crucial, as it can provide an opportunity for early treatment that may help prevent further progression of the disease. Neuropathology is currently considered the gold standard for diagnosing the presence and progression of neurodegenerative diseases. However, this is usually based on an autopsy performed after the death of a patient. Therefore, medical researchers are searching for an effective, non-invasive diagnostic method that can be employed for the early detection of neurodegeneration. The diagnosis of ALS is primarily based on symptoms, clinical examinations, and a series of tests to rule out other diseases. Owing to the complexity of the neurons behind observable or describable symptoms, it typically takes 9–15 months to diagnose ALS from the time when medical attention is required. Recent studies have shown that genetics can be widely used to study neurodegeneration. Several potential biomarkers (*SOD1*, *DAO*, *C9orf72*, *TARDBP*, *FUS*, *SETX*, *ANG*, *HNRNPA1*, *ALS2*, *VAPB*, *FIG4*, *OPTN*, *UBQLN2*, *VCP*, *MAPT*, *PFN1*) have been reported for the diagnosis of ALS, using either genetic or proteomic approaches [5,6,7,8,9]; however, none of these markers are robust enough to be applied in clinical practice. Currently, there is a lack of ALS biomarkers that can effectively assist in the diagnosis and detection of ALS.

Although many discoveries have been made in ALS research over the past two decades, the clinical pathogenesis of ALS remains unclear. In general, approximately 5–10% of ALS cases are familial ALS (fALS) and the remaining 90% are sporadic ALS (sALS) [10]. Although the heritability of neurodegenerative diseases is 40–80% [11,12,13,14], a significant fraction of the variance is non-hereditary, and there is strong evidence that environmental exposure is an important contributor to the disease pathogenesis [15]. Thus, the etiology of ALS is complex and involves both genetic and environmental factors. However, the specific molecular mechanisms underlying its pathogenesis remain poorly understood. The identification of epigenetic modifications associated with ALS may provide insights into the disease’s etiology.

Epigenetics is the study of gene expression or cell function through certain mechanisms or pathways without altering DNA sequences [16], including DNA methylation, histone modification, nucleosome repositioning, and post-transcriptional regulation by micro-RNAs. Such regulatory mechanisms alter the expression of genes that are closely related to the physiological and pathological states of individuals and can contribute to our understanding of human growth and disease development. DNA methylation refers to the addition of a methyl moiety to the cytosine-5 position to form 5-methylcytosines, mostly in genomic regions with high-frequency CpG sites called CpG islands [17]. In the human genome, approximately 70% of promoter regions contain CpG islands [18,19]. Therefore, abnormal genomic methylation may lead to various diseases. Increasing evidence has shown that alterations in DNA methylation can lead to differences in neurodegeneration and other brain diseases. Significant associations have been reported between DNA methylation in the brain/blood and Alzheimer’s disease (AD) [20,21,22,23], Parkinson’s disease (PD) [24,25], and ALS [26,27]. Therefore, DNA methylation analysis may help us explore the pathogenesis of ALS and identify appropriate biomarkers, shortening the diagnostic duration to guide subsequent ALS treatments.

In this study, we focused on designing a biomarker screening mechanism and identifying effective DNA methylation biomarkers for ALS, in addition to investigating the occurrence and patterns of methylation alterations during ALS progression. We proposed an approach to identify DNA methylation biomarkers, a traditional differential DNA methylation analysis incorporating associated comorbidity patterns and disease–gene associations. Electronic medical record (EMR) data from different medical databases were analyzed for comorbidity patterns in patients with ALS, and disease-specific associated genes were identified from the associated comorbidities. By integrating biomarkers primarily identified by differential DNA methylation analysis and cross-validating secondary significant disease genes from historical EMR comorbidity pattern analysis, we designed and screened effective biomarkers for ALS diagnosis. These results will enable the early detection of highly diverse pathogenic mechanisms in different patients with ALS and may provide effective and appropriate treatments to prolong survival.

## 2. Materials and Methods

**Figure 1:**
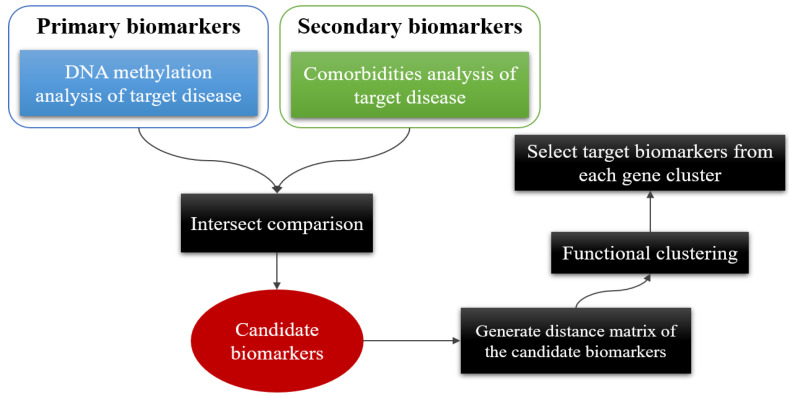
Flow chart showing the pipeline of exploring functional representative biomarkers by integrating primary biomarkers from DNA methylation analysis and secondary biomarkers from related comorbidity patterns.

Here, we proposed an efficient screening mechanism to identify novel biomarkers that can aid in the diagnosis of ALS by combining DNA methylation profiling and comorbidity patterns with gene functional analysis to investigate the phenomena and patterns of methylation during the progression of ALS and gain insights into the etiology of the disease. The processing stages of the entire method are shown in Figure 1, and the details of each step are described in the following sections.

### 2.1. Differential DNA Methylation Analysis for Primary Biomarkers

**Figure 2:**
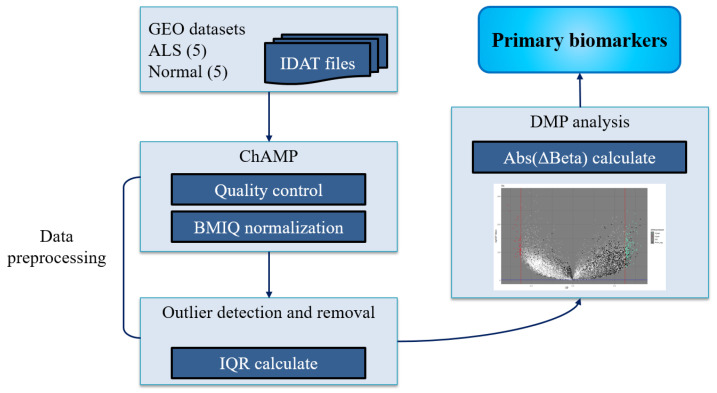
Pipeline operations to discover primary DMP biomarkers by using GEO methylation profiling data.

The GEO (Gene Expression Omnibus) database [28] is a public, functional genomic data repository. It is a sub-database of the NCBI database that specializes in storing high-throughput sequencing data submitted by the research community. To identify the primary biomarkers of methylation loci with significant differential expression, we downloaded an ALS genome-wide methylation profiling dataset (Illumina Human Methylation 450 K Bead Chip Array) from the GEO database (accession ID: GSE89472). This dataset collected blood methylation profiling data from five monozygotic twin pairs, with a total of 10 samples, including five ALS samples and five normal samples, for primary biomarker analysis (Figure 2). The first step in identifying differentially methylated DNA probes (DMPs) as ALS pathogenesis-associated biomarkers is to remove poor quality and/or unsuitable probes. Both ALS and normal control groups were integrated and analyzed through the Chip Analysis Methylation Pipeline (ChAMP) [29]. The standard ChAMP processes included champ.load, champ.QC, and champ.norm functions for methylation data profile preparation, and the champ.DMP function was applied to discover all differentially methylated positions. This is a standard pipeline package for quality control and data normalization. As the resolved methylation distributions differed, a standard normalization procedure was required. The BMIQ normalization technique provided by ChAMP was applied for each data entry. We then calculated the interquartile range (IQR) and defined the range between (Q1 −1.5 IQR) and (Q3 +1.5 IQR) to remove any outliers for each probe. After outlier removal, representative methylation levels of each probe in the experimental and control groups were confirmed. Using parameter settings on differential beta values (Abs(ΔBeta) ≥ 0.05) and applying multiple testing corrections using the Benjamini and Hochberg method to decrease the false discovery rate [30], a set of DMPs could be identified as primary biomarker candidates.

### 2.2. Comorbidity Analysis for Secondary Biomarkers

The initial symptoms of patients with ALS are quite different, and the disease progression varies from person to person. Not all patients with ALS experience the same symptoms or patterns of complications. However, progressive muscle weakness and paralysis are common. We analyzed the comorbidity patterns of ALS to identify specific comorbidity patterns for ALS and other mimic ALS diseases from the accumulated comorbidity patterns. Thereafter, disease–gene associations could be revealed and applied as comorbidity biomarkers for enhancing ALS-specific biomarker discovery.

The MIMIC-IV database (Medical Information Mart for Intensive Care IV (MIMIC-IV) database used in this study contains the inpatient medical records of Beth Israel Deaconess Medical Center (BIDMC) in the United States from 2008 to 2019 [31]. Two disease coding systems (ICD-9-CM and ICD-10-CM) were used during the data collection period. Therefore, data preprocessing was required to convert ICD-9-CM codes into the corresponding ICD-10-CM codes using the General Equivalence Mappings (GEMS) table, which was downloaded from the official website. If the ICD-9-CM in the GEMS table has no corresponding ICD-10-CM code; then, the ICD code will be ignored. If the corresponding situation is one too many, all ICD-10-CM codes remain. If an ICD-9-CM did not appear in the GEMS table, we searched for the nearest common ancestor from its upper level according to the tree structure of the ICD code. Finally, all the disease records were considered using only the first three ICD-10-CM codes in the following analyses.

Word2vec is a model for learning word vectors or word embeddings and was proposed by Tomas Mikolov et al. in 2013 [32]. This study applied the concept of word2vec to realize that the occurrence of a target disease is related to other comorbid diseases that occur together. In medicine, comorbidity refers to a physical and psychological personal history or the simultaneous presence of one or more medical conditions that coexist with the primary disease in a patient. Although it is currently known that some primary diseases are associated with comorbidities, many associations between primary disease and comorbidities are not clearly understood. Comorbidity is used to describe any disease that exists or occurs during the target disease development. It is primarily used to assess the severity and effect of comorbidities. Therefore, using comorbidity analytics, this research method was based on the CBOW of word2vec in the GenSim function library to predict diseases similar to ALS. Each inpatient medical record of a patient over 20 years of age in the MIMIC-IV database was applied to discover diseases with a high correlation with ALS.

Associated comorbidities could also be identified using the statistical method of case–control epidemiological studies. The ratio between the odds of developing the disease in the experimental and control groups was calculated to obtain the premise for controlling interference factors. First, the earliest onset of ALS was limited to those older than 20 years within the MIMIC-IV and the National Health Insurance Database (NHIRD) of Taiwan [33], and the diagnosed records were only considered as comorbidities within three years before the onset time. Patients with ALS with ages in the defined IQR intervals for male and female participants were evaluated in this study. Finally, the subjects who satisfied the constraints were selected as ALS samples in the experimental group. The case–control matching group was constructed by randomly sampling subjects (five times) without an ALS diagnosis according to age and sex. Diseases were considered statistically significant comorbidities by setting the odds ratio (OR), *p*-value of the chi-square test, and disease support rate. Because electronic health record databases usually contain only clinical observation information, this study utilized disease and gene associations supported by the DisGeNET database (SQLite2020-v7.0 version) [34].

### 2.3. Functional Clustering and Functional Annotation for Candidate DMPs

Each selected associated gene played a different role in the different functional pathways. Therefore, functionally representative candidate biomarkers can be selected by analyzing the correlations between gene functions. To identify appropriate methylation biomarkers, we used the gene functional annotation database, Gene Ontology (GO), for gene clustering analysis [35]. GO is a collection of defined functional annotation terms that are frequently applied in gene function analytics. Three main ontologies, including biological processes, molecular functions, and cellular components, were connected by GO terms in a directed acyclic graph (DAG) with a tree structure. In this study, functional clustering was based on candidate DMPs generated by cross-validating epigenetic and comorbidity biomarkers. Functional clustering provides an efficient and effective method for selecting representative biomarkers from each clustered functional group. This model calculates the distance between any two DMPs according to the defined GO terms for each DMP and their corresponding locations in GO tree structures. A distance matrix for all candidate DMPs was established as the input for subsequent clustering analysis.

#### 2.3.1. Pairwise Gene Distance Calculation

We combined the information content score [36] and Czekanowski–Dice distance [37] to define the distance between two GO terms. Thus, the distance between the two GO terms *t_i_* and *t_j_* is defined by Equation (1). dist(*t_i_*, *t_j_*) represents the distance between two GO terms, and dist_CD_(*t_i_*, *t_j_*) and dist_IC_(*t_i_*, *t_j_*) represent the Czekanowski–Dice distance and the information content score, respectively.

(1) $$\mathrm{dist}\left(t_{i},t_{j}\right)=\frac{{\mathrm{dist}}_{\mathrm{CD}}\left(t_{i},t_{j}\right)+{\mathrm{dist}}_{\mathrm{IC}}\left(t_{i},t_{j}\right)}{2}$$

The information content score in this study is an application of information theory to GO. From the structure of GO, we know that the closer a node is to the upper layer, the greater the variety of features it contains, and the more general and ambiguous the information. In contrast, a node closer to the lower layer contains more specific information. First, we defined the weight of each GO term as the basis of its information content. The weight of each GO term is a proportional coefficient defined as the number of genes annotated by specific GO terms divided by the total number of non-repetitive genes annotated by all GO terms, as described in Equation (2).

(2) $$W\left(t_{i}\right)=\frac{\mathrm{number}\;\mathrm{of}\;\mathrm{genes}\;\mathrm{annotated}\;\mathrm{by}\;\mathrm{the}\;\mathrm{GO}\;\mathrm{term}\;(G_{t_{i}})}{\mathrm{total}\;\mathrm{number}\;\mathrm{of}\;\mathrm{all}\;\mathrm{annotated}\;\mathrm{genes}\;}$$

We determined the information content score for the two GO terms by calculating the weight differences between their lowest common ancestors (LCA) and the two GO terms. Because more than one LCA may exist within a GO DAG structure, the LCA with the nearest distance was considered as the representative LCA in this study. If two GO terms were not located within the same GO tree ontology, they initially shared no common ancestors. In this case, we defined the normalized dist*_IC_* as one. This formula is defined in Equation (3).

(3) $${\mathrm{dist}}_{\mathrm{IC}}\left(t_{i},t_{j}\right)=\left\{\begin{array}{c} 2\times W\left(t_{LCA_{i,j}}\right)-W\left(t_{i}\right)-W\left(t_{j}\right)\\ 1\;\left(\mathrm{if}\;t_{LCA_{i,j}}\;\mathrm{not}\;\mathrm{exist}\right) \end{array}\right.$$

Another method, the Czekanowski–Dice distance, utilizes the overlapping relationship between two GO terms to calculate the distance, which refers to the number of genes shared between two GO terms. As shown in Equation (4), #*Gt_i_*Δ*Gt_j_* represents the number of exclusive genes annotated by *t_i_* or *t_j_*. The #*Gt_i_*∩*Gt_j_* and #*Gt_i_*∩*Gt_j_* represent the number of genes found in the union and intersection of the two assigned GO terms. When the number of exclusive genes between these two GO terms of *t_i_* and *t_j_* is high, the distance of dist_CD_ between *t_i_* and *t_j_* is relatively large.

(4) $${\mathrm{dist}}_{\mathrm{CD}}\left(t_{i},t_{j}\right)=\frac{\#G_{t_{i}}\#G_{t_{j}}}{\#G_{t_{i}}\cup G_{t_{j}}+\#G_{t_{i}}\cap G_{t_{j}}}$$

Once every two GO term distances are defined, the pairwise gene distance can be obtained by taking the average of all mutual GO term pair distances from the two target genes and their corresponding annotated GO terms.

#### 2.3.2. Functional Clustering of Candidate DMPs

After the candidate biomarker distance matrix was obtained, a clustering algorithm was applied to construct a clustered biomarker structure. Through cluster analysis, candidate DMPs can be categorized into different functional groups based on the calculated gene distances, which means that DMPs with highly similar functions can be classified in the same group. This study adopted an unweighted paired-group method using the arithmetic means (UPGMA) clustering algorithm to construct a biomarker tree structure. According to the definition of this algorithm, the two closest subgroups are merged into a higher-level cluster. Here, we applied GO function annotations for distance calculation so that closer genes would have a higher probability of being grouped into the same cluster. In other words, candidate biomarkers with similar functions were more likely to be categorized into the same group.

#### 2.3.3. Functional Annotation of Candidate DMPs

In addition, the functional annotation analysis tool provided by The Database for Annotation, Visualization and Integrated Discovery (DAVID) bioinformatics database [38] was applied to identify biological pathways in the KEGG database regarding differential methylation status. DAVID is a powerful functional gene analysis platform that helps users obtain comprehensive biological functional annotation information from a large set of genes or proteins. In a biological pathway, some genes are inhibitors and others are facilitators, with different functions depending on whether the genes are located upstream or downstream of the pathway. Therefore, we obtained biological pathway information of candidate DMPs annotated within KEGG from DAVID and analyzed the upstream and downstream locations of these DMPs to discover significantly correlated biological pathways and strengthen the selection of ALS biomarkers.

## 3. Results

### 3.1. Primary Biomarkers from Differentially Methylated Positions (DMPs)

The DNA methylation profiles provided by five ALS patients and five normal subjects from the GEO database were compared to identify major DMP biomarkers. By setting the adjusted *p*-value (<0.05), 3881 statistically significant probe sites were retrieved. Among them, 204 DMPs with significant adjusted *p*-values were identified by using the parameter setting on beta value difference ≥ 0.05 (Abs(ΔBeta) ≥ 0.05). These 204 DMP loci corresponded to 128 genes, respectively, of which 20 DMPs are located within gene promoter regions.

Since the methylation biomarkers selected in this study were expected to design a methylation-specific qPCR commercial toolkit in the future, DMP analysis was considered the key analysis in this study. In addition, we also performed differential methylation region (DMR) analysis by using the champ.DMR() function in the ChAMP package. However, the significant methylation loci of ALS patients used in this study possessed only 3881 loci after data preprocessing, and there is no significant DMR gene could be found from these twin samples under various parameter settings.

### 3.2. Comorbidity Biomarkers from EMR Datasets

Comorbidity biomarkers were obtained from two different medical datasets: the MIMIC-IV from the USA and the NHIRD from Taiwan. First, two medical datasets were analyzed using the CBOW model of word2vec technology, and the cosine similarity of each disease in the vector space was calculated to determine the set of diseases that were highly correlated with ALS. Each dataset was trained 10 times with the CBOW model, and the top 15 diseases most similar to ALS were retrieved according to the cosine similarity scores. Finally, the top ten most frequently appearing diseases were selected as representatives to identify the corresponding disease genes based on the correlation between diseases and genes in DisGeNET. In total, 2973 annotated genes were associated with the top 10 ALS-similar disease groups from MIMIC-IV. In addition, 2091 annotated genes were associated with ALS-related disease groups in the NHRID. The detailed disease names of the 10 disease groups with corresponding similarities in these two datasets are provided in Supplementary Table S1.

### 3.3. Cross-Verification and Gene Functional Clustering Results

The primary epigenetic biomarkers obtained from the ALS genome-wide methylation profiling dataset were then cross-compared with comorbidity biomarkers, ALS-associated disease genes identified from the two medical datasets (MIMIC-IV and NHIRD), and two different approaches. In other words, we cross-compared epigenetic biomarkers with comorbidity biomarkers detected in two different EMR datasets based on the word2vec technique and a statistical assessment approach. The results showed that 16 genes could be identified between epigenetic biomarkers and comorbidity-related genes based on the word2vec technique from the MIMIC-IV datasets and 10 genes from the NHIRD. In more detail, based on word2vec technique approach in MIMIC-IV, we could discover 16 associated genes including *MPZ*, *HOMER1*, *MGLL*, *SOD3*, *CACNA1H*, *ERBB4*, *TBCD*, *DPYSL2*, *ANGPT1*, *DEAF1*, *ITGAX*, *KIF7*, *SEMA3E*, *SLC6A3*, *BHMT*, and *DBT*; while using word2vec approach in NHIRD dataset, we could identify 10 genes, including *MPZ*, *HOMER1*, *MGLL*, *SOD3*, *CACNA1H*, *ERBB4*, *TBCD*, *DPYSL2*, *ANGPT1*, and *VCL*. Based on a cross-comparison of statistical assessment methods with epigenetic biomarkers, we could identify 20 associated genes from the MIMIC-IV, including *MPZ*, *HOMER1*, *MGLL*, *SOD3*, *CACNA1H*, *ERBB4*, *TBCD*, *DPYSL2*, *ANGPT1*, *DEAF1*, *ITGAX*, *KIF7*, *EMA3E*, *SLC6A3*, *ZP3*, *MDN1*, *EIF4A3*, *RIPK2*, *C1R*, and *SORL1*. Again, based on the statistical assessment approach, 12 genes could be identified from the NHIRD dataset, including *MPZ*, *HOMER1*, *MGLL*, *SOD3*, *CACNA1H*, *ERBB4*, *TBCD*, *DPYSL2*, *ANGPT1*, *RHOBTB2*, *ADAMTS1*, and *UNC5D*. The genes associated with epigenetic biomarkers and comorbidities from the two medical datasets and two different approaches are shown in Supplementary Tables S2 and S3. As a few disease groups were not annotated with any corresponding disease genes using DisGeNET, only comorbidities with corresponding gene annotations are shown in these Supplementary Materials. The results from the two different approaches were further verified by considering the methylation biomarkers located within specific genetic regions. Finally, only nine methylation biomarker candidates, including *MPZ*, *HOMER1*, *MGLL*, *SOD3*, *CACNA1H*, *ERBB4*, *TBCD*, *DPYSL2*, and *ANGPT1* were identified using previously described processes.

**Figure 3:**
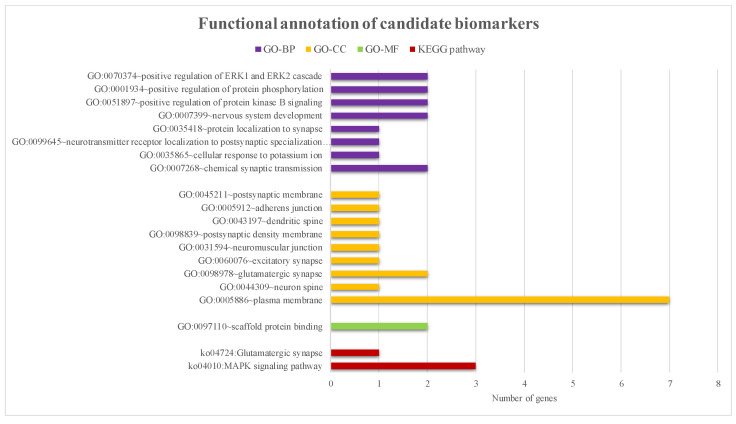
Functional annotation results of the nine candidate biomarkers. The horizontal axis represents the number of genes corresponding to each function. The purple, yellow and green legends were the corresponding annotation results of candidate biomarkers in three main ontologies: biological processes, cellular components, and molecular functions. The red legend represents the annotation results of KEGG biological pathways.

To design genetic testing toolkits that can effectively assist in early ALS diagnosis from liquid biopsies, it is helpful to select functionally representative biomarkers by analyzing associations through gene functions. According to the nine screened methylation biomarkers, the corresponding GO function annotations are shown in Figure 3. All of them are highly associated with ALS. For example, as noted in the results for GO:0007268 (chemical synaptic transmission) and GO:0099645 (neurotransmitter receptor localization to postsynaptic specialization membrane) within the GO biological process category, there were some studies reported that synaptic transmission was significantly associated with ALS neurological function [39]. In addition, excitotoxicity or neuronal cell death caused by excessive intracellular calcium levels due to overstimulation of the excitatory neurotransmitter glutamate was thought to be a common mechanism in all forms of ALS [3]; such instructions were also presented in the GO cellular component annotations. These discovered GO annotations in the three category trees and corresponding relationships were applied to calculate their functional distances between gene pairs. The UPGMA clustering algorithm then used the obtained functional distances to automatically cluster the nine candidates into three groups: (*SOD3*), *(CACNA1H*), *(MPZ*, *HOMER1*, *MGLL*, *ERBB4*, *TBCD*, *DPYSL2*, and *ANGPT1).* Genes with similar functions were grouped into clusters. Biomarkers in each functional group were sorted according to the degree of difference in methylation, and one gene from each functional group was selected as the representative biomarker. The combination of *SOD3*, *CACNA1H*, and *ERBB4* was finally selected from the three clusters and was considered the best combination of methylation biomarker testing kits for ALS detection.

In addition, to enhance the selection of ALS biomarker candidates, nine candidate biomarkers were annotated using KEGG pathways. It was found that these candidate genes were also significantly associated with ko04010 (MAPK signaling pathway) and ko04724 (Glutamatergic synapse) in the KEGG biological pathway dataset (As shown in the red legend of Figure 3). The MAPK signaling pathway is a fundamental mitogen/stress-activated signaling pathway that regulates cell proliferation, differentiation, survival, and death and is considered to be significantly related to cancer. In recent years, many studies have implicated cellular stress in ALS conditions, such as ER stress, DNA damage, oxidative stress, and growth factor depletion, leading to severe damage to several cellular processes, including protein homeostasis, RNA metabolism, and vesicle transport. The MAPK signaling pathway is associated with various ALS-related cellular pathophysiological defects [40]. Another annotated glutamatergic synaptic pathway is the main excitatory synapse pathway in the brain. These synapses are composed of glutamate located within the presynaptic vesicles and glutamate receptors on the postsynaptic membrane. According to research, synaptic disorders or dysfunctions may underlie many developmental and degenerative neurological diseases [41]. Elevated glutamate concentrations in the synapses induce excitotoxicity, and their downstream effects are considered a common pathogenesis of ALS [42]. In summary, if the functional groups are clustered and associated with various ALS pathogenic mechanisms, it would provide outstanding detection that is not only beneficial for early detection but also facilitates better treatment suggestions for medical doctors.

## 4. Discussion and Conclusions

DNA methylation is an important molecular mechanism underlying human diseases. This study aimed to explore an efficient and effective screening mechanism for methylation biomarker selection, particularly for the early detection of ALS through liquid biopsy tests. We identified potential biomarkers for ALS through a comprehensive analysis by investigating blood DNA methylation differences between patients with ALS and healthy controls, and significant comorbid disease genes from two different medical datasets (MIMIC-IV and NHIRD). The epigenetic biomarkers identified by traditional methylation profiling data and comorbidity biomarkers annotated by EMRs may share a common mechanism for further exploration and selection of biomarkers. The results of gene–disease association analysis revealed that “hereditary and idiopathic peripheral neuropathy (ICD-9: 356)”, “nerve root and plexus disorders (ICD-9: 353)”, “aphagia and dysphagia (ICD-10: R13), and “respiratory failure (ICD-10: J96)” were commonly associated with ALS symptoms. In addition, there are reports showing that vocal cord dysfunction is associated with the manifestation of ALS [43], with symptoms including hoarseness, hypophonia, dry cough at night, episodes of inspiratory stridor, and dyspnea. Dyspnea is a common and well-known symptom in patients with ALS, and stridor is a common symptom of multiple system atrophy that may occasionally occur in other neurodegenerative diseases [44,45,46]. However, vocal cord dysfunction has received little attention in the ALS-related literature. The identified significant comorbid disease genes are considered important secondary biomarkers for subsequent clinical validation.

The proposed method identified three functionally representative methylation biomarkers, *SOD3*, *CACNA1H*, and *ERBB4*, which have all been reported to be significantly associated with ALS and may be useful for the early diagnosis and subsequent precision treatment of ALS. Mutations in *SOD3* have been linked to several diseases, including familial amyloidotic polyneuropathy type 1, diabetes, and hypertension, and are risk factors for atherosclerosis [47]. Studies in previous years have also shown that mutations or overexpression of *SOD3* may be associated with ALS [48]. For the second gene, previous studies have found that missense mutations in *CACNA1H* produced minor but significant changes in T-type channel activity, consistent with a loss of channel function. This finding indicates that *CACNA1H* is a susceptibility gene for ALS [49,50]. Additionally, *ERBB4* mutations led to reduced autophosphorylation of *ERBB4* upon neuregulin-1 (NRG-1) stimulation, resulting in clinically affected individuals with both upper and lower motor neuron involvement and a lack of obvious cognitive dysfunction. This study suggests that disruption of the neuregulin-*ERBB4* pathway is involved in ALS pathogenesis [51]. The publication of these studies provided additional support for our findings.

In this study, nine candidate methylation biomarkers were found to apply to clinical databases in different regions using two different cross-referencing approaches. However, one study reported that the clinical characteristics of ALS differ among various ethnic groups [52]. From the cross-comparison results (shown in Supplementary Tables S2 and S3), it can be seen that among the intersecting genes with significant comorbidities, *VCL*, *RHOBTB2*, *ADAMTS1*, and *UNC5D* were unique to the NHIRD dataset, whereas *ZP3*, *DEAF1*, *MDN1*, *KIF7*, *SLC6A3*, *ITGAX*, *EIF4A3*, *RIPK2*, *C1R*, *SORL1*, *SEMA3E*, *BHMT*, and *DBT* were identified in the United States MIC-IV dataset only. It can be inferred that genetic differences between Asian and USA ALS patients with ALS may contribute to the differences in clinical characteristics among different ethnic groups.

In summary, the current unmet medical needs in the ALS market include curative drugs, earlier diagnosis, identification of specific biomarkers associated with ALS, and extended patient survival with improved symptoms. Among these issues, early diagnosis by utilizing appropriate biomarkers is the most important challenge. By comprehensively analyzing the GEO methylation profiling data and EMR databases, we identified a set of significant and representative methylation biomarkers for the detection of ALS. In addition, by combining ALS comorbidity datasets from different racial groups, we identified biomarkers that may be exclusively applicable to different ethnic groups. These new findings provide promising evidence that DNA methylation biomarkers can be used for the early diagnosis of ALS patients.

## Data Availability

The datasets used in the current study are available as follows: All Gene Expression Omnibus (GEO) datasets are available on the platform GPL13534 (https://www.ncbi.nlm.nih.gov/geo/query/acc.cgi?acc=GPL13534, accessed on 1 September 2024) and the MIMIC-IV database (Medical Information Mart for Intensive Care-IV) (https://physionet.org/content/mimiciv/2.2/, accessed on 1 September 2024). However, the datasets are from the NHIRD and were used only for this study under policies limitation. Therefore, the data is not publicly available. Interested researchers can obtain the data through the formal application (https://nhird.nhri.org.tw/en/, accessed on 1 September 2024).

## Supplementary Materials

The following supporting information can be downloaded at: https://www.mdpi.com/article/10.3390/bioengineering11101020/s1. Table S1: Calculation of the cosine similarity for each disease in the MIMIC-IV and NHIRD datasets using the CBOW model of word2vec technology for the top 10 highly correlated disease groups with ALS; Table S2: Identified associated comorbidity disease genes from two different approaches (word2vec and statistical odds ratio) between MIMIC-IV and DNA methylation primary biomarkers from ALS epigenomic samples; Table S3: Identified associated comorbidity disease genes from two different approaches (word2vec and statistical odds ratio) between NHIRD and DNA methylation primary biomarkers from ALS epigenomic samples.
