# Supplementary material for: An Effective DNA Methylation Biomarker Screening Mechanism for Amyotrophic Lateral Sclerosis (ALS) Based on Comorbidities and Gene Function Analysis
Source: Bioengineering (Basel). 2024 Oct 12;11(10):1020. doi: 10.3390/bioengineering11101020 (PMC11505182; doi:10.3390/bioengineering11101020)
Supplement: Supplementary file 1 [file bioengineering-11-01020-s001.zip › Supplementary Table S1.pdf]

Supplementary Table S1. Calculation of the cosine similarity for each disease in the MIMIC-IV and NHIRD datasets using the CBOW model of word2vec technology for the top 10 highly correlated disease groups with ALS.

MIMIC-4		NHIRD	
**ICD-10-CM**	**Disease name**	**ICD-9-CM**	**Disease name**
Q32	Congenital malformations of trachea and bronchus	V46	Other dependence on machines and devices
Q31	Congenital malformations of larynx	934	Foreign bodies in trachea bronchus and lung
Q07	Other congenital malformations of nervous system	933	Foreign bodies in pharynx and larynx
J38	Diseases of vocal cords and larynx	737	Curvature of spine
G80	Cerebral palsy	519	Other diseases of respiratory system
G23	Other degenerative diseases of basal ganglia	359	Muscular dystrophies and other myopathies
G11	Hereditary ataxia	343	Infantile cerebral palsy
F72	Severe intellectual disabilities	139	Late effects of other infectious and parasitic diseases
Q02	Microcephaly	994	Effects of other external causes
Q05	Spina bifida	326	Late effects of intracranial abscess or pyogenic infection
