# Supplementary material for: An Effective DNA Methylation Biomarker Screening Mechanism for Amyotrophic Lateral Sclerosis (ALS) Based on Comorbidities and Gene Function Analysis
Source: Bioengineering (Basel). 2024 Oct 12;11(10):1020. doi: 10.3390/bioengineering11101020 (PMC11505182; doi:10.3390/bioengineering11101020)
Supplement: Supplementary file 1 [file bioengineering-11-01020-s001.zip › Supplementary Table S2.pdf]

Supplementary Table S2. Identified associated comorbidity disease genes from two different approaches (word2vec and statistical odds ratio) between MIMIC-IV and DNA methylation primary biomarkers from ALS epigenomic samples; the grey grid indicates genes in the DisGenet database that are annotated as being associated with the diseases (ICD-10-CM).

		**ALS**	**W2V(TOP10)**					
Gene name	***beta_diff***	***G12***	***J38***	***G23***	***Q02***	***G80***	***Q05***	***F72***
MPZ	-0.058103059							
HOMER1	0.0500659							
MGLL	-0.091682367							
SOD3	-0.052781241							
CACNA1H	-0.051671094							
ERBB4	-0.092216964							
TBCD	0.055823477							
DPYSL2	-0.054262751							
ANGPT1	-0.072167322							
DEAF1	-0.071295549							
ITGAX	-0.051517017							
KIF7	-0.055397657							
SEMA3E	-0.098907746							
SLC6A3	-0.070583327							
BHMT	-0.061916558							
DBT	0.052108121							

		**ALS**	**OR comorbidity**				
Gene name	***beta_diff***	***G12***	***R13***	***R62***	***C82***	***E46***	***R47***
MPZ	-0.058103059						
HOMER1	0.0500659						
MGLL	-0.091682367						
SOD3	-0.052781241						
CACNA1H	-0.051671094						
ERBB4	-0.092216964						
TBCD	0.055823477						
DPYSL2	-0.054262751						
ANGPT1	-0.072167322						
DEAF1	-0.071295549						
ITGAX	-0.051517017						
KIF7	-0.055397657						
SEMA3E	-0.098907746						
SLC6A3	-0.070583327						

ZP3	-0.060435033						
MDN1	0.056886275						
EIF4A3	-0.089896602						
RIPK2	-0.050872126						
C1R	-0.067458677						
SORL1	-0.090807461						
