# Supplementary material for: An Effective DNA Methylation Biomarker Screening Mechanism for Amyotrophic Lateral Sclerosis (ALS) Based on Comorbidities and Gene Function Analysis
Source: Bioengineering (Basel). 2024 Oct 12;11(10):1020. doi: 10.3390/bioengineering11101020 (PMC11505182; doi:10.3390/bioengineering11101020)
Supplement: Supplementary file 1 [file bioengineering-11-01020-s001.zip › Supplementary Table S3.pdf]

Supplementary Table S3. Identified associated comorbidity disease genes from two different approaches (word2vec and statistical odds ratio) between NHIRD and DNA methylation primary biomarkers from ALS epigenomic samples; the grey grid indicates genes in the DisGenet database that are annotated as being associated with the diseases (ICD-9-CM).

		**ALS**	**W2V(TOP10)**
Gene name	***beta_diff***	***335***	***359***
MPZ	-0.058103059		
HOMER1	0.0500659		
MGLL	-0.091682367		
SOD3	-0.052781241		
CACNA1H	-0.051671094		
ERBB4	-0.092216964		
TBCD	0.055823477		
DPYSL2	-0.054262751		
ANGPT1	-0.072167322		
VCL	-0.072211199		

		**ALS**	**OR comorbidity**			
Gene name	***beta_diff***	***335***	***356***	***354***	***722***	***728***
MPZ	-0.058103059					
HOMER1	0.0500659					
MGLL	-0.091682367					
SOD3	-0.052781241					
CACNA1H	-0.051671094					
ERBB4	-0.092216964					
TBCD	0.055823477					
DPYSL2	-0.054262751					
ANGPT1	-0.072167322					
RHOBTB2	-0.064946839					
ADAMTS1	-0.064411414					
UNC5D	-0.057662729					
